# Frailty combined with nutritional risk for predicting stroke-associated pneumonia: a cohort study based on a nomogram model

**DOI:** 10.3389/fneur.2026.1771608

**Published:** 2026-07-31

**Authors:** Kailibinuer Aimaier, Jiarui Xiong, Chunrui Liu, Shuhong Zhou, Guangwei Liu, Feng Li

**Affiliations:** 1 Department of Neurology & Nursing, The First Affiliated Hospital of Chongqing Medical University, Chongqing, China; 2 College of Culture and Tourism, Chongqing Business Vocational College, Chongqing, China

**Keywords:** frailty, nutrition, pulmonary infection, risk prediction, stroke

## Abstract

**Introduction:**

Stroke-associated pneumonia (SAP) is a common and serious complication in patients with acute severe stroke, and existing risk assessment tools have limited predictive accuracy in critically ill populations. This study innovatively incorporated frailty and nutritional risk, which reflect stress tolerance and overall physiological reserve, into an early SAP prediction model.

**Methods:**

A retrospective cohort study was conducted on 293 critically ill stroke patients admitted to the Neurocritical Care Unit of the First Affiliated Hospital of Chongqing Medical University between 2013 and 2024. Collect clinical characteristics and laboratory indicators of patients, assess their frailty status and nutritional risk, and analyze the additive interaction effect between the two on the occurrence of SAP. Independent predictors were identified through multivariate logistic regression and incorporated into a visual nomogram. Model performance was evaluated using the area under the receiver operating characteristic curve (AUC), calibration plots, decision curve analysis, and 10-fold cross-validation.

**Results:**

A total of 293 patients with severe stroke were included in this study, among whom 126 (43%) developed SAP. The results of the additive interaction analysis showed a positive additive interaction between frailty and nutritional risk in the development of SAP, with an attributable proportion (AP) of 0.711 (95% CI = 0.358 ~ 1.065) and a synergy index (SI) of 3.694 (95% CI = 1.200 ~ 11.364). A SAP risk prediction model incorporating age, nasogastric tube use, neutrophil-to-lymphocyte ratio (NLR), frailty status, and nutritional risk demonstrated good discriminative performance, with an area under the curve (AUC) of 0.848, which was significantly higher than that of the conventional SAP prediction score (ISAN score: AUC = 0.589). Internal validation showed that the model achieved an accuracy of 73.93%, sensitivity of 77.30%, and specificity of 71.95%, indicating good stability. The calibration curve demonstrated good agreement between predicted and observed outcomes. Decision curve analysis (DCA) indicated that the model provided substantially greater clinical net benefit than the ISAN score.

**Discussion:**

This study is the first to integrate frailty and nutritional risk into an SAP prediction model, significantly improving early risk identification and providing an innovative, practical tool for precision prevention and targeted intervention in critically ill stroke patients.

## 1 Introduction

Stroke is one of the leading causes of death and disability worldwide, with its high incidence and associated burden posing significant public health challenges (1, 2). Stroke-associated pneumonia (SAP) is a common and severe infectious complication in patients with stroke, defined as pneumonia developing within 7 days of stroke onset in non-mechanically ventilated individuals (3). Reported incidence rates of SAP range from 5.3 to 37.9% (4), accounting for 10.1 to 37.3% of stroke-related deaths (5). The presence of SAP increases the risk of in-hospital mortality by approximately 40% ~ 45% (6). Due to its insidious early symptoms, SAP is often associated with delayed diagnosis and intervention, which substantially affects the prognosis of critically ill stroke patients (7). Therefore, early monitoring and risk stratification of SAP are of critical clinical importance.

Previous studies have established several strong predictors of SAP, including advanced age, impaired consciousness, dysphagia, stroke severity, and systemic inflammatory responses (3, 4, 8, 9). Although several predictive tools, such as the Acute Ischemic Stroke-Related Pneumonia Risk Score (age, atrial fibrillation, dysphagia, sex, stroke severity, A2DS2) and the Integer-Based Pneumonia Score(prestroke independence, sex, age, National Institutes of Health Stroke Scale, ISAN), have been developed for SAP risk assessment, they are primarily limited to routinely collected clinical and laboratory indicators (10–12). These tools fail to accurately capture patients’ overall physiological reserve and metabolic status, leading to limited predictive performance. Consequently, there is an urgent need to develop comprehensive indicator systems that reflect frailty, immunological function, and metabolic status to improve the accuracy of early SAP identification.

Frailty and nutritional risk represent critical dimensions of general physiological function and metabolic reserve, with potential implications for SAP development (13, 14). Frailty manifests as multisystem functional decline and diminished stress tolerance, increasing susceptibility to infection through impaired immunity, muscle weakness, and dysphagia (15). Nutritional risk impairs immunological and metabolic functions (16). Malnutrition can lead to immunosuppression and impaired tissue repair, thereby increasing the risk of infection (17). Moreover, frailty and nutritional risk are interrelated and mutually reinforcing (18); their coexistence often indicates heightened physiological vulnerability and may synergistically increase SAP risk (19). However, systematic research examining their combined predictive role in SAP remains limited, particularly lacking a multifactorial, integrated quantitative prediction model for patients with severe stroke.

Against this backdrop, this study aims to evaluate the predictive value of frailty status and nutritional risk at admission for SAP in patients with acute severe stroke. Based on these findings, a comprehensive risk prediction model was constructed. By integrating frailty, nutritional status, and conventional clinical indicators, this study aims to facilitate early quantitative risk assessment for SAP. This approach will provide scientific evidence for individualized clinical interventions and precision prevention and control, while offering an innovative tool for the early management of patients with severe stroke.

## 2 Patients and methods

### 2.1 Study design

This retrospective cohort study utilized electronic medical record data from the First Affiliated Hospital of Chongqing Medical University. The study population comprised critically ill stroke patients admitted to the Neurological Intensive Care Unit (NICU) from January 2013 to December 2024. The study report follows the Transparency Reporting of Individual Prognosis or Diagnosis Multivariable Prediction Models (TRIPOD) statement.

### 2.2 Setting and participants

Inclusion criteria were: (1) Stroke diagnosed by imaging according to the Chinese Guidelines for the Diagnosis and Treatment of Acute Ischemic Stroke 2023 (20) or the Chinese Guidelines for the Diagnosis and Treatment of Cerebral Hemorrhage (2019) (21); (2) Age ≥18 years; (3) First episode, onset ≤48 h, and NICU stay ≥48 h. The exclusion criteria were: (1) Pre-existing pulmonary infection or community-acquired pneumonia prior to stroke onset; (2) Use of immunomodulatory agents within 48 h of admission; (3) Presence of mechanical ventilation, hemodynamic instability, multiple organ failure, severe hepatic or renal dysfunction, immune system disorders, or malignancy at admission; (4) Loss to follow-up or missing clinical data.

**Figure 1:**
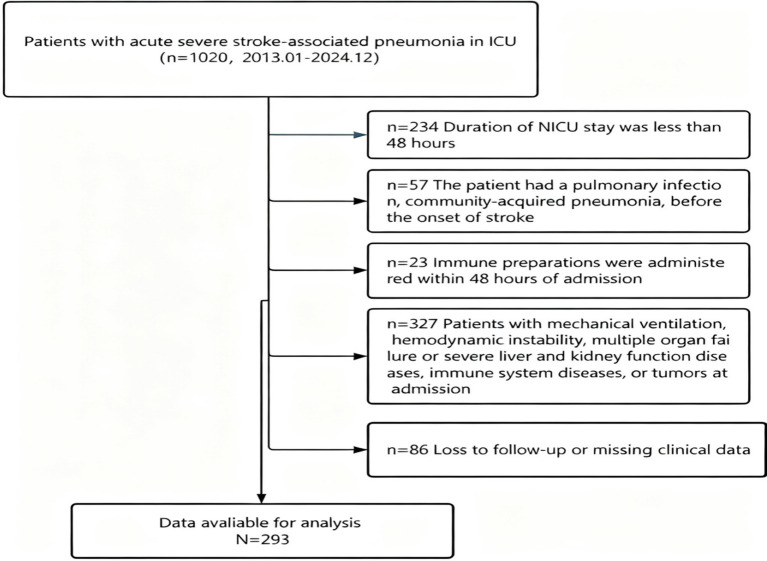
Flow chart of study subjects screening ICU, Intensive Care Unit; NICU, Neurological Intensive Care Unit.

Sample size calculation: Sample size was determined based on the EPV (events per variable) principle for multivariate logistic regression models (22). Each variable requires at least 10 outcome events, with a maximum of 10 variables in the model; at least 100 outcome events would be required. Based on literature reporting a SAP incidence of approximately 38% in stroke patients (4) and accounting for 10% ineligible samples, the calculation indicated a minimum requirement of 290 patients. Ultimately, 293 patients were enrolled. The subject selection flowchart is shown in Figure 1.

### 2.3 Data collection

#### 2.3.1 Screening of potential predictive variables

By retrieving studies from PubMed, Embase, Web of Science, MedLine, and The Cochrane Library databases (2005–2024) related to SAP risk factors, combined with clinical indicators available in the electronic medical record system of the Department of Neurology, Chongqing Medical University Affiliated First Hospital, 26 candidate variables potentially influencing SAP occurrence were ultimately identified for model construction.

#### 2.3.2 Data collection

Patient information was retrospectively extracted from the electronic medical record system of the Neurological Intensive Care Unit at the First Affiliated Hospital of Chongqing Medical University. (1) General Information: Including age, sex, smoking status, alcohol consumption, past medical history (hypertension, diabetes, coronary heart disease, atrial fibrillation), nasogastric tube status. (2) Disease characteristics: stroke type, stroke location (for patients with ischemic stroke, the infarct location was recorded; for patients with hemorrhagic stroke, the hemorrhage location was recorded), Acute Physiology and Chronic Health Evaluation II(APACHE II), National Institute of Health Stroke Scale(NIHSS), Glasgow Coma Scale(GCS), modified Rankin scale(mRS). (3) Laboratory biochemical indicators: Albumin(ALB), Hemoglobin(HB), Platelet Count(PLT), Neutrophil-to-Lymphocyte Ratio(NLR), Serum White Blood Cell Count(WBC). (4) Swallowing Function Assessment: Swallowing function was evaluated using both retrospective clinical record review and the Kubota Water Swallow Test. Dysphagia was defined as a physician-documented diagnosis of swallowing dysfunction or a Kubota Water Swallow Test result of Grade III or above (abnormal). Assessments were performed by trained healthcare professionals according to standardized clinical procedures. (5) Frailty and Nutritional Risk Assessment: Trained neurologists with extensive clinical experience assessed frailty status and nutritional risk using the Clinical Frailty Scale(CFS) and Modified Nutrition Risk in the Critically Ill Score(mNUTRIC) based on patients’ electronic medical records. Assessors were blinded to the study outcome measures. CFS < 5 indicated no frailty; CFS ≥ 5 indicated frailty. mNUTRIC < 5 indicated low risk; mNUTRIC ≥ 5 indicated high risk. The CFS was independently scored by two trained investigators following a standardized protocol, and inter-rater reliability was assessed. The results showed excellent agreement between the two raters [weighted Kappa = 0.949 (95% CI = 0.929 ~ 0.970), *p* < 0.001], indicating a high level of consistency between the two evaluators.

### 2.4 Outcome definition

The primary outcome is the occurrence of SAP during NICU hospitalization. SAP diagnosis is based on modified Centers for Disease Control and Prevention (CDC) criteria: meeting any one systemic criterion (fever ≥38 °C, abnormal WBC count, or altered mental status in patients ≥70 years) and any two respiratory criteria (new purulent sputum or increased sputum production, new or worsening cough/dyspnea/respiratory distress, pulmonary rales or bronchial breath sounds, gas exchange impairment), along with new or progressive infiltrates, consolidation, or ground-glass opacities on chest imaging (23, 24).

### 2.5 Traditional predictive scores

The Integer-Based Pneumonia Score, prestroke independence, sex, age, and National Institutes of Health Stroke Scale (ISAN) was developed by Smith et al. in 2015 based on the UK population. It includes sex, age, NIHSS score, and pre-stroke disability status (25). Age (<60 years: 0 points; 60–69 years: 3 points; 70–79 years: 4 points; 80–89 years: 6 points; ≥90 years: 8 points); Sex (Female: 0 points; Male: 1 point); NIHSS score (0–4 points: 0 points; 5–15 points: 4 points; 16–20 points: 8 points; ≥21 points: 10 points); Modified Rankin Scale score (<4 points: 0 points; ≥4 points: 2 points).

### 2.6 Missing values

Patients with missing data rates >20% were considered lost to follow-up. Variables with missing data exceeding 20% (C-reactive protein, high-sensitivity C-reactive protein, procalcitonin) were excluded from this study. No other variables had missing data, and no imputation methods were applied,resulting in a total of 23 variables included in the final analysis.

### 2.7 Statistical analysis

Statistical analysis was performed using SAS9.4 and R4.5.2. Quantitative data meeting normal distribution requirements were expressed as mean ± standard deviation (*x* ± s), with intergroup comparisons conducted using Student’s t-test. Quantitative data that did not meet normal distribution requirements were reported as median (interquartile range) [M(P25, P75)], and intergroup comparisons were conducted using the Mann–Whitney U test. Qualitative data were presented as frequencies and percentages (%). Intergroup comparisons were performed using the Chi-square test. Additive interaction between frailty status and nutritional risk was assessed using the “non-frailty and low nutritional risk” group as the reference category. The interaction contrast ratio (ICR), attributable proportion due to interaction (AP), and synergy index (SI) were calculated. In single-factor analyses, variables with *p* < 0.05 were entered into a LASSO-logistic regression model. Multicollinearity among independent variables was evaluated using the variance inflation factor (VIF), VIF < 5 indicated no significant multicollinearity. A multivariable logistic regression model was used to assess the independent and joint effects of frailty status and nutritional risk on the occurrence of SAP, with odds ratio (OR) and 95% confidence interval (CI) reported. Based on the regression results, a nomogram prediction model was constructed using the rms package in R software. Receiver operating characteristic (ROC) curves were plotted, and the area under the curve (AUC) was calculated to assess model performance. Calibration plots and decision curve analysis (DCA) curves were generated to evaluate model calibration and clinical utility. Internal validation was performed using 10-fold cross-validation.

## 3 Results

### 3.1 Baseline characteristics of severe stroke patients and univariate analysis of factors influencing concurrent SAP

Among 293 severe stroke patients, 126 (43%) developed SAP during hospitalization. The prevalence rates were as follows: neither frailty nor high nutritional risk (173, 59.04%), frailty alone (31, 10.58%), high nutritional risk alone (36, 12.29%), and concurrent frailty and high nutritional risk (53, 18.09%). Patients with SAP differed significantly from those without SAP in age, alcohol consumption, concomitant atrial fibrillation, nasogastric tube, frailty, nutritional risk, and APACHE II scores (*p* < 0.05). The univariate analysis results for baseline characteristics and factors influencing SAP in severe stroke patients are shown in Table 1.

**Table 1:** Baseline characteristics and univariate analysis of SAP risk factors in severe stroke patients.

Variable/Category	SAP		*t*/*Z*/*χ*^2^	*p*
	No (*n* = 167)	Yes (*n* = 126)		
General characteristics				
Sex [*n* (%)]				
Female	38 (22.75)	37 (29.37)	1.648	0.199
Male	129 (77.25)	89 (70.63)		
Age [*n* (%)]				
<65 years old	45 (26.95)	8 (6.35)	20.562	<0.001
≥65 years old	122 (73.05)	118 (93.65)		
Smoking [*n* (%)]				
No	105 (62.87)	86 (68.25)	0.916	0.339
Yes	62 (37.13)	40 (31.75)		
Alcohol [*n* (%)]				
No	108 (64.67)	95 (75.40)	3.882	0.049
Yes	59 (35.33)	31 (24.60)		
Comorbidities				
Hypertension [*n* (%)]				
No	58 (34.73)	40 (31.75)	0.287	0.592
Yes	109 (65.27)	86 (68.25)		
Diabetes [*n* (%)]				
No	122 (73.05)	89 (70.63)	0.209	0.648
Yes	45 (26.95)	37 (29.37)		
Atrial fibrillation [*n* (%)]				
No	155 (92.81)	98 (77.78)	13.774	<0.001
Yes	12 (7.19)	28 (22.22)		
Coronary heart disease [*n* (%)]				
No	129 (77.25)	87 (69.05)	2.491	0.114
Yes	38 (22.75)	39 (30.95)		
Nasogastric tube [*n* (%)]				
No	11 2(67.07)	43 (34.13)	31.272	<0.001
Yes	55 (32.93)	83 (65.87)		
Disease characteristics				
Stroke type [*n* (%)]				
Hemorrhagic	41 (24.55)	26 (20.63)	0.624	0.429
Ischemic	126 (75.45)	100 (79.37)		
Infarction location [*n* (%)]				
Brainstem infarction	19 (15.08)	11 (11.00)	0.806	0.369
Basal ganglia infarction	65 (51.59)	40 (40.00)	3.009	0.083
Cortical infarction	81 (64.29)	72(72.00)	1.517	0.218
Subcortical infarction (internal capsule/corona radiata)	40 (31.75)	27(27.00)	0.602	0.438
Cerebellar infarction	21 (16.67)	7 (7.00)	4.800	0.028
Hemorrhage location [*n* (%)]				
Intraventricular hemorrhage	13 (31.71)	0 (0.00)	10.229	<0.001
Brainstem hemorrhage	21 (51.22)	11 (42.31)	0.506	0.477
Basal ganglia hemorrhage	9 (21.95)	7 (26.92)	0.216	0.642
Thalamic hemorrhage	8 (19.51)	10(38.46)	2.908	0.088
Lobar hemorrhage	1 (2.44)	1 (3.85)	<0.001	0.999
Cerebellar hemorrhage	2 (4.88)	3 (11.54)	/	0.369
Subarachnoid hemorrhage	12 (29.27)	6 (23.08)	0.310	0.577
NHISS score[x ± s]	15.74 ± 7.85	15.37 ± 6.76	0.425	0.671
APACHE II score[x ± s]	16.49 ± 5.85	17.89 ± 6.21	−1.981	0.049
GCS score[x ± s]	8.53 ± 3.88	8.30 ± 3.37	0.521	0.603
mRS score[M(P25, P75)]	4 (4.5)	4 (4.5)	0.733	0.463
Dysphagia [*n* (%)]				
No	141 (84.43)	108 (85.71)	0.093	0.761
Yes	26 (15.57)	18 (14.29)		
Nutritional risk [*n* (%)]				
Low nutritional risk	148 (88.62)	56 (44.44)	66.275	<0.001
High nutritional risk	19 (11.38)	70 (55.56)		
Frailty status [*n* (%)]				
No	146 (87.43)	63 (50.00)	49.188	<0.001
Yes	21 (12.57)	63 (50.00)		
Frailty status and nutritional risk [*n* (%)]				
No frailty with low nutritional risk	133 (79.64)	40 (31.75)	80.358	<0.001
Frailty with low nutritional risk	15 (8.98)	16 (12.70)		
No frailty with high nutritional risk	13 (7.78)	23 (18.25)		
Frailty with high nutritional risk	6 (3.59)	47 (37.30)		
Laboratory biochemical parameters				
PLT[10^9^/L,x ± s]	186.05 ± 73.27	179.40 ± 61.90	0.840	0.402
NLR[M(P25, P75)]	10.70 (6.00, 17.10)	12.60 (7.70, 17.10)	1.957	0.050
WBC[10^9^/L,x ± s]	11.84 ± 4.83	12.71 ± 3.78	−1.737	0.083
Alb [g/L,x ± s]	38.84 ± 6.82	39.24 ± 6.45	−0.508	0.612
HB[g/L,x ± s]	129.99 ± 24.79	129.71 ± 22.46	0.102	0.919
Conventional prediction score				
ISAN score	6.00 (4.00, 6.00)	6.00 (5.00, 7.00)	2.532	0.011

SAP, stroke-associated pneumonia; APACHE II, Acute Physiology and Chronic Health Evaluation II score; NIHSS, National Institutes of Health Stroke Scale; GCS, Glasgow Coma Scale; mRS, modified Rankin Scale; ALB, albumin; Hb, hemoglobin; PLT, platelet count; NLR, neutrophil-to-lymphocyte ratio; WBC, white blood cell count; ISAN, integer-based pneumonia risk score; “/”: no statistical test was performed (Fisher’s exact test).

### 3.2 Additive interaction between frailty and nutritional risk on SAP

**Figure 2:**
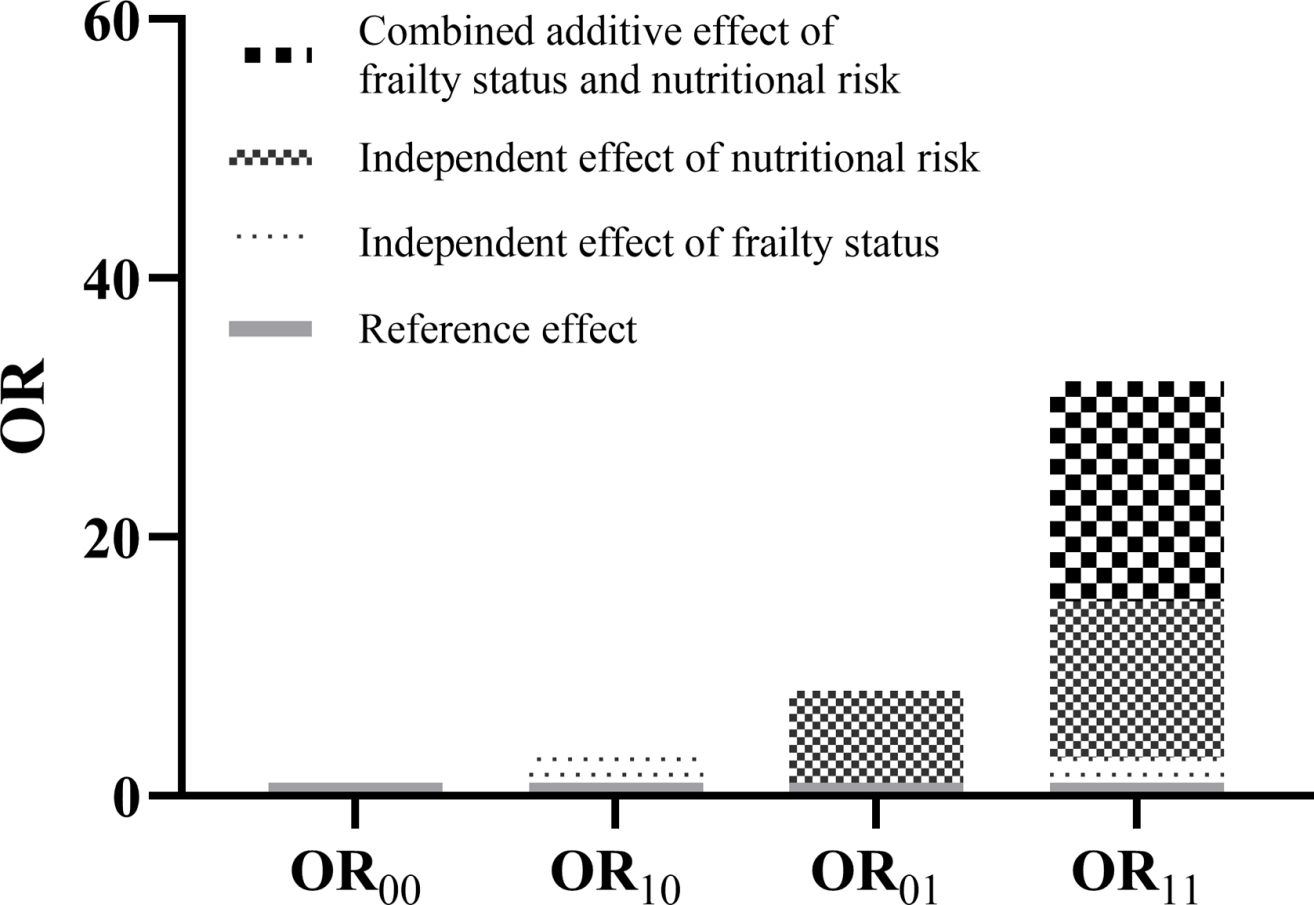
Histogram illustrating the additive interaction effect between frailty status and nutritional risk on SAP. *OR*, Odds ratio. The *x*-axis represents the effect estimates of frailty status and nutritional risk (*OR*00: Non-frailty with low nutritional risk; *OR*10, Frailty with low nutritional risk; *OR*01, Non-frailty with high nutritional risk; *OR*11, Frailty with high nutritional risk); the *y*-axis represents the odds ratios (*OR*s) for the risk of stroke-associated pneumonia (SAP).

Using the group without frailty and low nutritional risk as the reference category, the effects of frailty and nutritional risk on SAP were further examined. Patients with concurrent frailty and high nutritional risk exhibited the highest incidence of SAP (88.68%, 47/53), with an OR of 29.089(95% CI = 10.470 ~ 80.814, *p* < 0.001). The attributable proportion due to interaction (AP) was 0.711 (95% CI = 0.358 ~ 1.065, *p* < 0.05), indicating that approximately 71.1% of SAP cases among patients with both frailty and high nutritional risk could be attributed to the interaction between these two factors. The synergy index (SI) was 3.694 (95% CI = 1.200 ~ 11.364, p < 0.05), suggesting that the joint effect of frailty and nutritional risk on SAP was approximately 3.694 times greater than the sum of their individual effects. The interaction contrast ratio (ICR) was 28.433 (95% CI = −11.411 ~ 68.278, *p* > 0.05), although this estimate did not reach statistical significance. Taken together, these findings support the presence of a positive additive interaction between frailty and nutritional risk in the development of SAP. The results are shown in Figure 2.

### 3.3 Selection of predictors for the SAP prediction model

**Figure 3:**
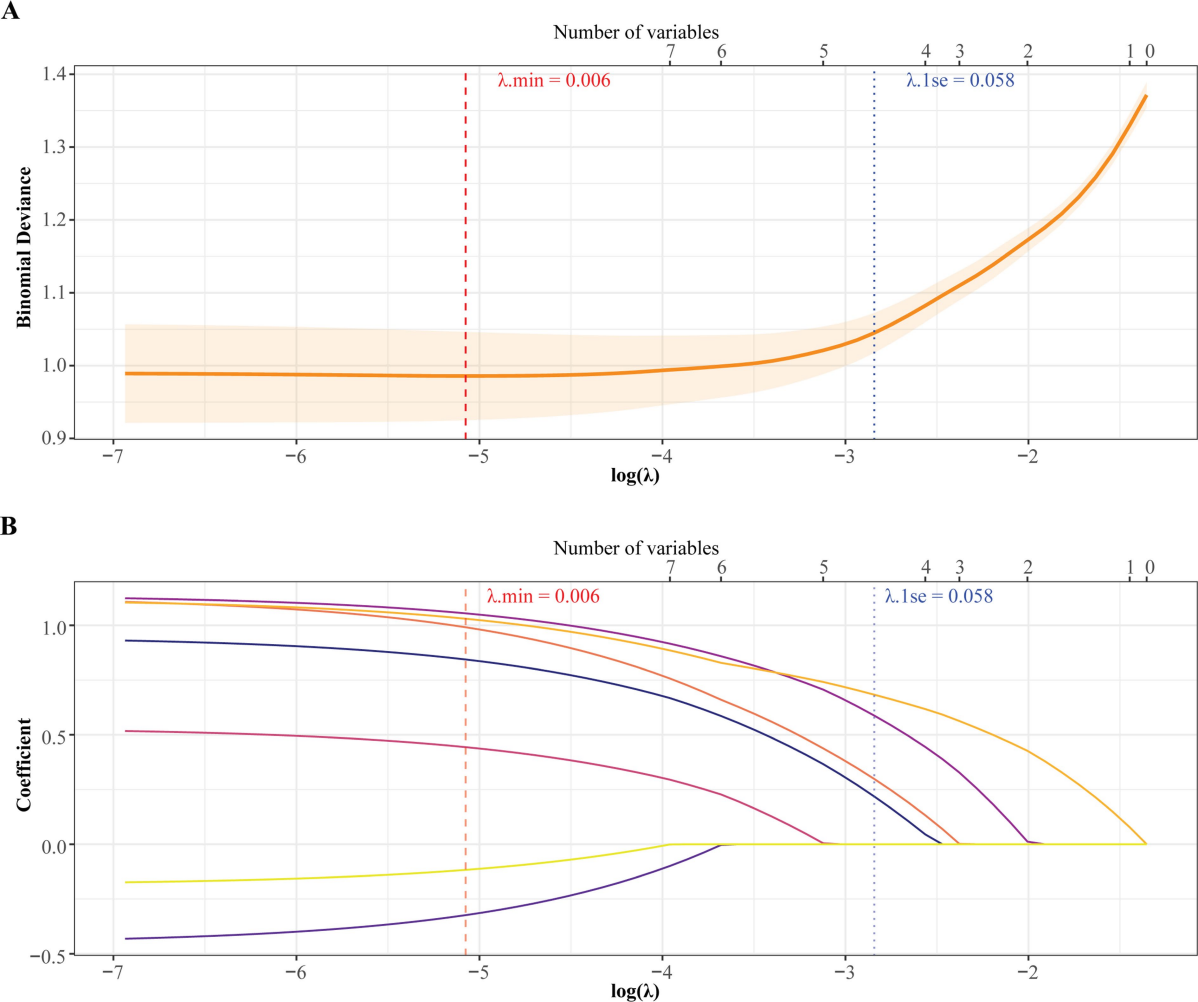
Screening results of the Lasso-logistic model. **(A)** Curve of binomial deviance versus log(*Λ*), **(B)** Curve of regression coefficients versus log(*λ*). **(A)** The binomial deviance plotted against log(*λ*). The blue dashed vertical line indicates the optimal *λ* value selected by cross-validation (*λ* within one standard error of the minimum). The red dashed vertical line indicates the *λ* value corresponding to the minimum cross-validated deviance. **(B)** Coefficient shrinkage paths of variables. The *x*-axis represents log(*λ*), and the *y*-axis represents the estimated regression coefficients. Each curve corresponds to one variable, and coefficients shrink toward zero as *λ* increases. In **B**, the curves from top to bottom correspond to nasogastric tube, frailty and nutritional status, age, NLR, alcohol consumption, atrial fibrillation, and APACHE II score. Variables with larger absolute coefficient values and later shrinkage toward zero contribute more importantly to model prediction.

Variables with *p* < 0.05 in the univariate analysis were entered into the LASSO logistic regression model, and the variable coding scheme is presented in Table 2. Based on the λ1se criterion, four predictors with non-zero coefficients were retained: age, nasogastric tube, frailty and nutritional risk status, and NLR (Figure 3).

**Table 2:** Value assignment of predictor variables.

Independent variable	Variable coding
Age	<65 years old = 0; ≥65 years old = 1
Alcohol consumption	No = 0; Yes = 1
Atrial fibrillation	No = 0; Yes = 1
Nasogastric tube	No = 0; Yes = 1
Frailty status and nutritional risk	Using “No frailty with low nutritional risk” as the reference category, dummy variables were set as follows: frailty with low nutritional risk = (Z1 = 1, Z2 = 0, Z3 = 0); No frailty with high nutritional risk = (Z1 = 0, Z2 = 1, Z3 = 0); frailty with high nutritional risk = (Z1 = 0, Z2 = 0, Z3 = 1)
NLR	Using “<6” as the reference category, dummy variables were constructed as follows: 6–25 = (Z1 = 1, Z2 = 0); >25 = (Z1 = 0, Z2 = 1)
APACHE II	Using “≤10” as the reference category, dummy variables were constructed as follows: 11–20 = (Z1 = 1, Z2 = 0); ≥21 = (Z1 = 0, Z2 = 1)

APACHE II, Acute Physiology and Chronic Health Evaluation II score; NLR, neutrophil-to-lymphocyte ratio.

### 3.4 Development and validation of the SAP prediction model in patients with severe stroke

**Figure 4:**
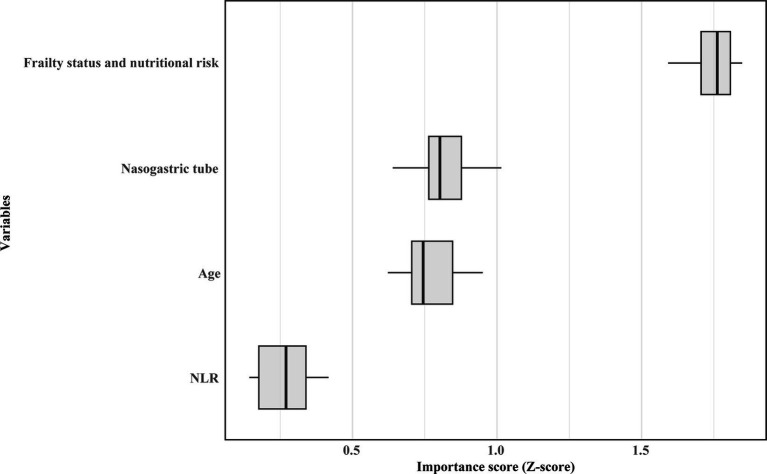
The ranking of predictor importance in the SAP predictive model. The *y*-axis represents the independent variables included in the prediction model, and the *x*-axis represents the variable importance scores. The boxplot displays the distribution of importance scores for each variable in the model. A higher importance score indicates a greater contribution of the variable to the prediction of stroke-associated pneumonia (SAP) risk. NLR: neutrophil-to-lymphocyte ratio.

The predictors selected by the LASSO logistic regression model were entered into a multivariable logistic regression model to develop the SAP prediction model (Table 3). All VIF values were below 2, indicating no evidence of multicollinearity. Predictor importance ranked, in descending order, as follows: frailty and nutritional risk status, nasogastric tube, age, and NLR (Figure 4). The SAP prediction model showed good discrimination, with an AUC of 0.848 (95% CI = 0.802 ~ 0.895). Calibration was satisfactory, with a Hosmer–Lemeshow *χ*^2^ of 12.612 (*p* = 0.126) and a Brier score of 0.150. Internal validation using 10-fold cross-validation demonstrated that, at a cutoff value of P_(SAP)_ = 0.410, the model achieved an accuracy of 0.739 (95% CI = 0.679 ~ 0.800), a sensitivity of 0.773 (95% CI = 0.681 ~ 0.865), and a specificity of 0.720 (95% CI = 0.603 ~ 0.836).

**Table 3:** Multivariate logistic regression analysis of SAP in patients with severe stroke.

Independent variable	*β*	SE	Wald	*p*	*OR* (95%CI)	VIF
Constant	−3.667	0.601	37.263	<0.001		
Age						
<65 years old					1.0 (reference)	
≥65 years old	0.980	0.454	4.648	0.031	2.664 (1.093,6.490)	1.087
Nasgastric tube						
No					1.0 (reference)	
Yes	1.168	0.301	15.079	<0.001	3.216 (1.784,5.800)	1.070
Frailty status and nutritional risk						
No frailty with low nutritional risk					1.0 (reference)	
Frailty with low nutritional risk	1.265	0.450	7.915	0.005	3.544 (1.468,8.556)	1.096
No frailty with high nutritional risk	1.749	0.420	17.334	<0.001	5.750 (2.524,13.101)	1.083
Frailty with high nutritional risk	3.247	0.530	37.552	<0.001	25.709 (9.101,72.628)	1.189
NLR						
<6					1.0 (reference)	
6 ~ 25	1.168	0.442	6.973	0.008	3.215 (1.351,7.649)	1.427
>25	2.132	0.617	11.931	0.001	8.435 (2.515,28.287)	1.421

NLR, neutrophil-to-lymphocyte ratio; VIF, variance inflation factor.

### 3.5 Comparison of the SAP prediction model with conventional prediction scores in patients with severe stroke

**Figure 5:**
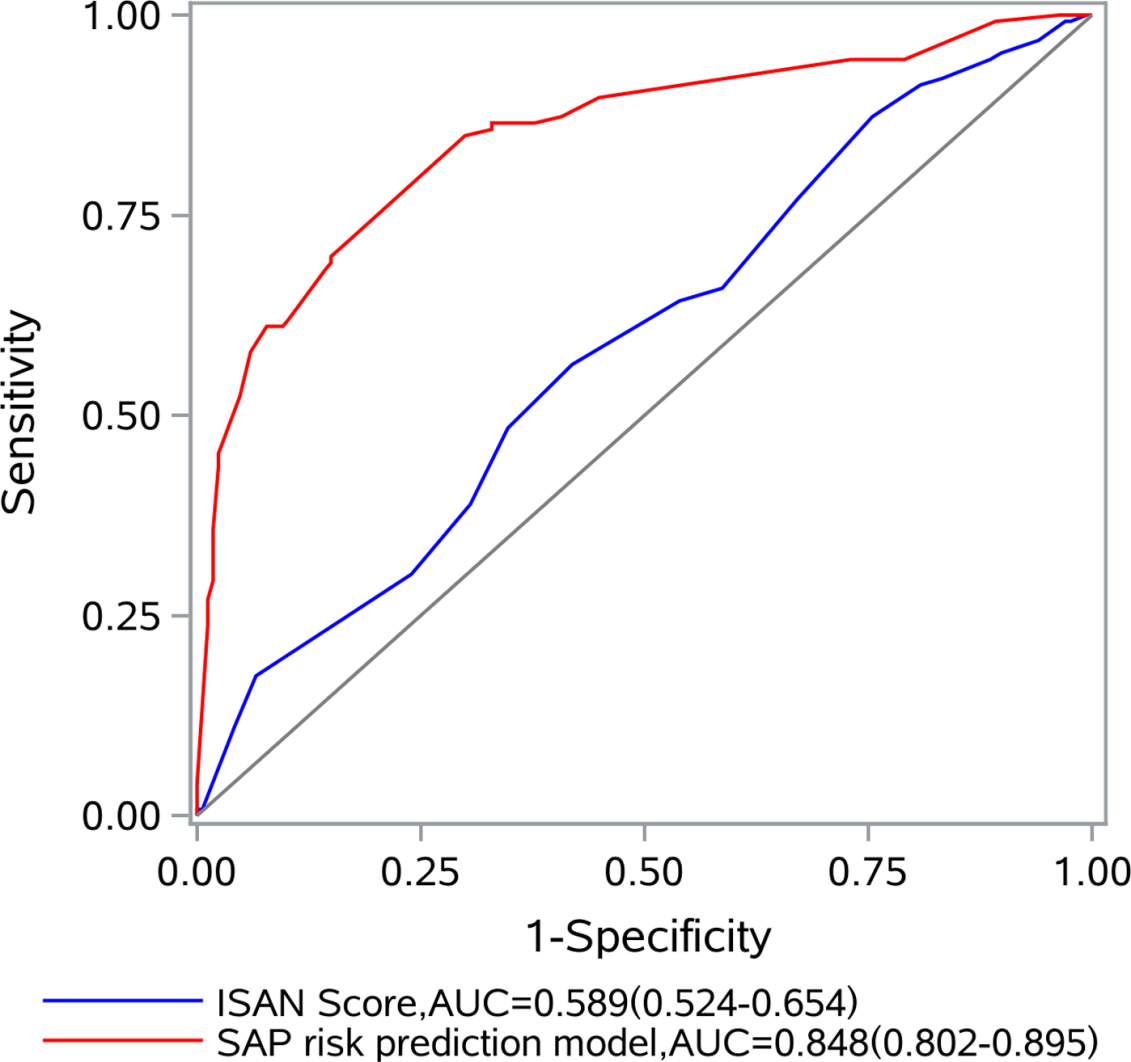
ROC curve comparison of SAP risk prediction models and scores in patients with severe stroke. The *x*-axis represents the false positive rate, and the *y*-axis represents the true positive rate. The diagonal line serves as a reference line (AUC = 0.5), indicating no discriminative ability. The blue curve represents the ISAN score, and the red curve represents the SAP prediction model. A curve farther away from the reference line indicates better discriminative performance of the model.

The predictive performance of the SAP prediction model and conventional SAP risk scores was compared. The SAP prediction model demonstrated superior discrimination, with an AUC of 0.848, which was higher than that of the conventional ISAN score (AUC = 0.589). The AUC of the SAP prediction model increased by 0.258 compared with the ISAN score (95% CI = 0.198 ~ 0.319, *p* < 0.001) (see Figure 5).

### 3.6 Nomogram development and validation of the SAP prediction model in patients with severe stroke

**Figure 6:**
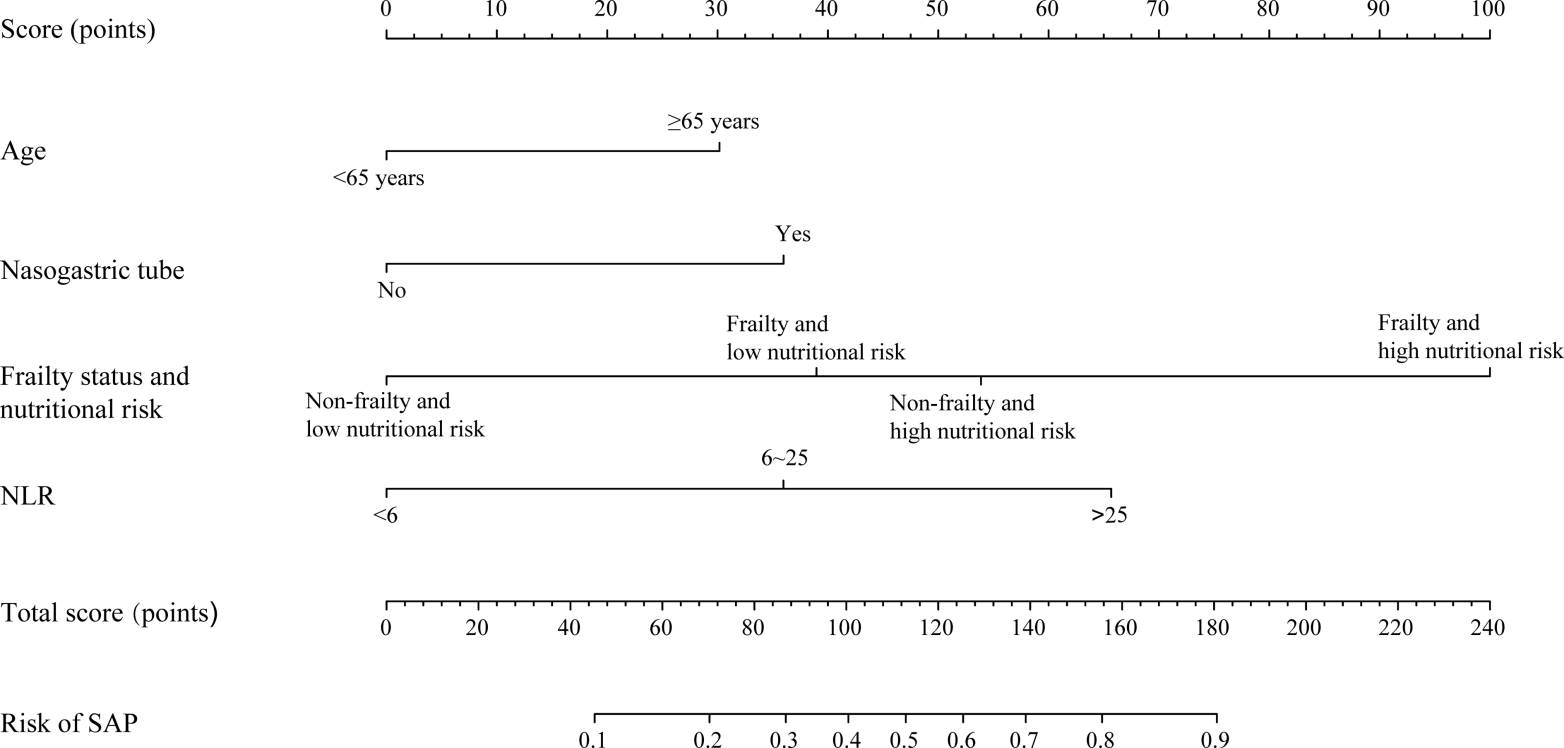
Nomogram model for predicting SAP. The *x*-axis represents the false positive rate, and the y-axis represents the true positive rate. The diagonal line serves as a reference line (AUC = 0.5), indicating no discriminative ability. The blue curve represents the ISAN score, and the red curve represents the SAP prediction model. A curve farther away from the reference line indicates better discriminative performance of the model.

**Figure 7:**
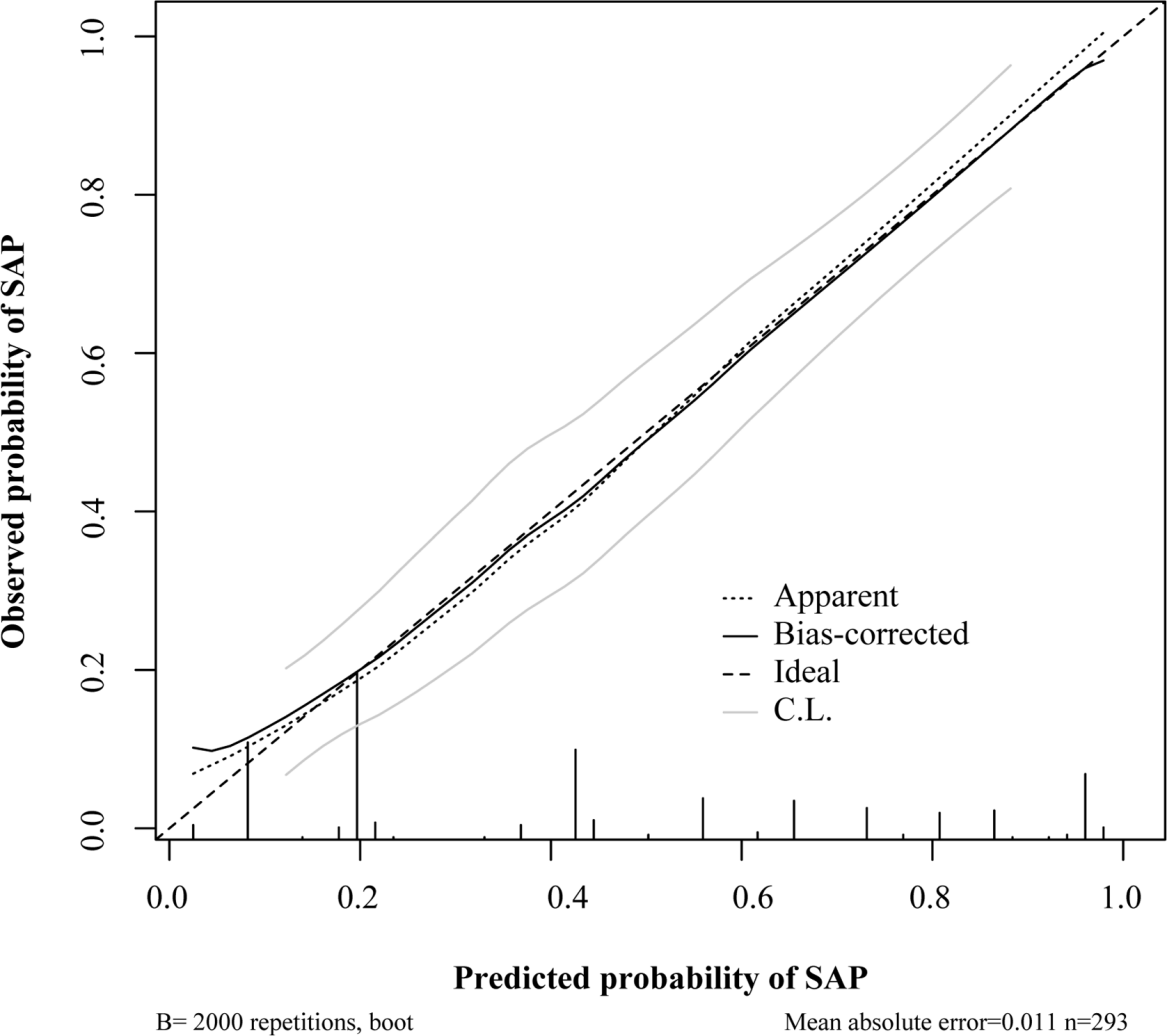
Calibration curve of the SAP predictive model. The *x*-axis represents the predicted probability of the event occurrence (range: 0–1), and the *y*-axis represents the observed probability of the event occurrence (range: 0–1). The gray dashed line indicates the ideal calibration line (perfect agreement between predicted and observed probabilities). The figure shows both the apparent (dotted line) and bias-corrected (solid line) calibration curves. The study included 293 samples (*n* = 293), and bias correction was performed using bootstrap resampling (*B* = 2,000). The overall mean absolute error was 0.011.

The SAP prediction nomogram is shown in Figure 6, and the corresponding point assignment is presented in Table 4. Internal validation with 2,000 bootstrap resamples showed that the nomogram achieved a C-index of 0.848 for predicting SAP, indicating good discrimination. The calibration curve demonstrated good agreement between the predicted and observed risks of SAP, with a mean absolute error of 0.011 (Figure 7).

**Table 4:** Scoring of predictors in the SAP nomogram model for prediction.

Predictors	Points
Age	
<65 years old	0
≥65 years old	30
Nasgastric tube	
No	0
Yes	36
Frailty status and nutritional risk	
No frailty with low nutritional risk	0
Frailty with low nutritional risk	39
No frailty with high nutritional risk	54
Frailty with high nutritional risk	100
NLR	
<6	0
6 ~ 25	36
>25	66

NLR, neutrophil-to-lymphocyte ratio.

### 3.7 Decision curve analysis and risk stratification based on the SAP prediction model in patients with severe stroke

**Figure 8:**
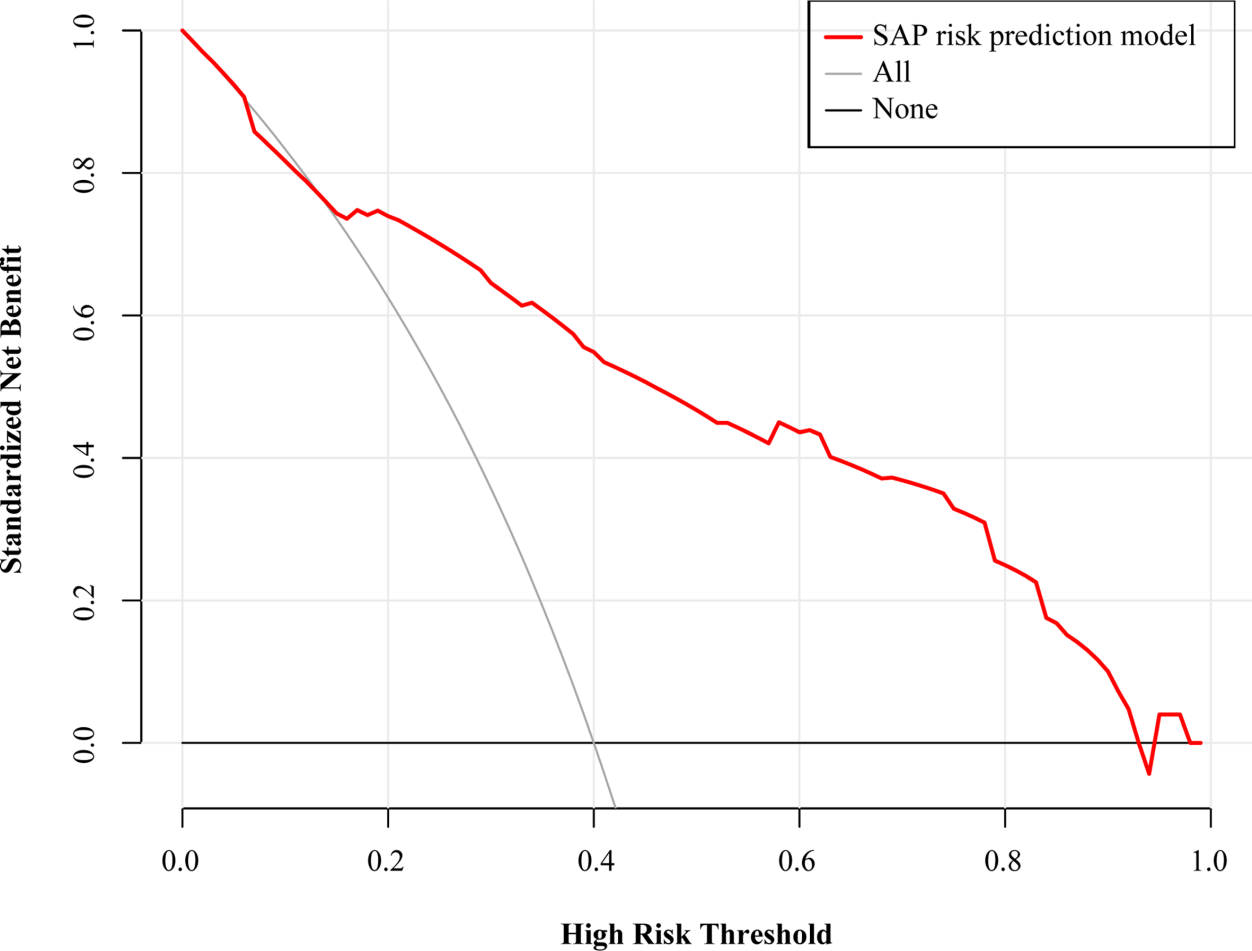
Decision curve analysis of the SAP predictive model. The SAP prediction model (red curve) represents decision-making based on the model developed in this study. The “all” strategy (light gray curve) represents the “treat-all” strategy, assuming that all patients are classified as high risk and receive intervention. The “none” strategy (black horizontal line) represents the “treat-none” strategy, assuming that no patients are classified as high risk and no intervention is performed.

The decision curve analysis demonstrated that the SAP prediction model achieved a higher net benefit than the treat-none strategy across a range of threshold probabilities, indicating potential clinical utility for guiding decision-making (Figure 8). Based on the nomogram-derived predicted risk of SAP, patients were stratified into low-risk, intermediate-risk, and high-risk groups. The observed incidence of SAP in the three risk groups was 14.05% (17/121), 40.51% (32/79), and 82.80% (77/93), respectively, with significant differences among the three groups (*p* < 0.05) (Table 5).

**Table 5:** Risk stratification of SAP prediction model.

SAP risk stratification	Nomogram score range	Risk range	Observed SAP incidence	*χ* ^2^	*p*
Low risk	0 ~ 69	<0.20	14.05% (17/121)	101.67	<0.001
Intermediate risk	70 ~ 124	0.20 ~ <0.60	40.51% (32/79)		
High risk	≥125	≥0.6	82.80% (77/93)		

SAP, stroke-associated pneumonia.

## 4 Discussion

This study included 293 critically ill stroke patients admitted to the NICU to investigate the predictive value of frailty and nutritional risk for the development of SAP. Results indicated that age, nasogastric tube, frailty and nutritional risk, as well as NLR were all significantly associated with the occurrence of SAP (p < 0.05). The individualized nomogram model constructed based on these findings demonstrated superior discrimination, calibration, and clinical net benefit compared to the ISAN score. The importance score of predictive factors showed that frailty and nutritional risk were the most important predictors in the model. These results indicate that frailty and nutritional risk independently play significant roles in predicting the development of SAP. Incorporating both factors into risk prediction models significantly enhances their discriminatory power and clinical utility, thereby enabling early diagnosis and individualized intervention for high-risk SAP patients. This comprehensive predictive model addresses the limitations of traditional scoring tools in reflecting patients’ overall physiological reserve and nutritional status, providing new quantitative evidence for early risk stratification and precise prevention in critically ill stroke patients.

Among the significant SAP risk factors identified in this study, frailty—as a comprehensive indicator representing impaired physiological reserves and reduced stress tolerance—demonstrated remarkable predictive value (26). The prevalence of frailty (28.7%) in this study closely aligns with findings reported by Burton et al. (14). Previous studies have indicated that frailty significantly increases the risk of pulmonary infection in elderly patients and that early identification and intervention can reduce infection rates (27, 28). However, previous research has primarily focused on pulmonary infections in non-neurocritical or general medical patients, with relatively few studies addressing SAP (29–31). This study further confirms that frailty is strongly associated with the development of SAP, implying that frailty may enhance vulnerability to infection via routes such as decreased immunological function, dysphagia, and malnutrition (14). Given the high prevalence of frailty among elderly stroke patients and its significant impact on SAP risk, incorporating frailty assessment into routine SAP risk evaluation and prevention strategies is of considerable clinical importance.

This study further identified nutritional risk as a significant predictor of SAP. Nutritional risk reflects the degree of imbalance between energy supply and demand, is strongly associated with impaired immune defense, dysphagia, and compromised mucosal barriers, thereby increasing stroke patients’ susceptibility to SAP (32). Jiang et al. found that stroke patients with poor nutritional status were more likely to have impaired swallowing reflexes and aspiration, thereby increasing the risk of pneumonia (33). Ciancarelli et al. reported that malnutrition reduces stroke patients’ resistance to pathogens by impairing cellular immunity and antioxidant defense mechanisms (17). Furthermore, ICU cohort studies have demonstrated that elevated nutritional risk scores are significantly correlated with infection incidence, frequency of mechanical ventilation, and mortality (13). Thus, early identification and individualized nutritional support for high-risk patients may reduce stroke-related infections and improve overall clinical outcomes.

Frailty and nutritional risk frequently coexist in stroke patients, both reflecting inadequate physiological reserves, impaired immune defense, and reduced stress tolerance. These conditions may synergistically contribute to the development of SAP (34). Previous studies in cardiovascular disease, perioperative surgery, and oncology populations have demonstrated that combined assessment of frailty and nutritional status more accurately predicts infections, postoperative complications, and adverse outcomes compared with single indicators (35–37). In this study, 18.1% of severe stroke patients exhibited concurrent frailty and high nutritional risk. Moreover, the interaction effect evaluation indicators further demonstrated that frailty and nutritional risk have a synergistic amplification effect on the risk of disease during the occurrence of SAP, implying that healthcare providers need to enhance the early monitoring and assessment of frailty and nutritional status in critically ill stroke patients. Based on this finding, the present study pioneered the integration of frailty, nutritional risk, and other significant clinical factors to construct a SAP nomogram model for individualized risk assessment. This nomogram not only visually illustrates the contribution of each predictor to outcomes, but it also has outstanding predictive performance (AUC = 0.856). Ten-fold cross-validation confirmed the stability of the model, while calibration curves and DCA further validated its clinical utility. This decision curve model enables clinicians to quickly assess SAP risk and conduct individualized risk stratification, providing a reference for identifying high-risk patients and developing prevention strategies (38, 39). Future research should further explore the interactive effects between frailty and nutritional risk, as well as combined intervention strategies, to achieve more precise infection risk management and improved overall outcomes for stroke patients.

Limitations: Several limitations of this study should be acknowledged. First, its single-center retrospective design and small sample size necessitate further validation in multicenter, large-scale populations. Second, this study excluded patients who were receiving mechanical ventilation at admission. Therefore, the findings are primarily applicable to stroke patients who were not mechanically ventilated, and their generalizability to critically ill patients requiring mechanical ventilation remains to be further evaluated. Third, while primarily evaluating the predictive value of frailty and nutritional risk at admission for SAP, prospective longitudinal studies are needed to explore the association between dynamic changes in these indicators and outcomes. Finally, although the nomogram model constructed in this study demonstrated good stability and predictive performance after internal validation, its stability and clinical generalizability require further validation in external cohorts.

## 5 Conclusion

Frailty and nutritional risk are significant independent predictors of SAP in patients with severe stroke. This study pioneers the integration of these factors into a comprehensive predictive model, enabling individualized risk quantification through a visual nomogram. The model has strong predictive accuracy and clinical value, providing a practical tool for precise assessment and individualized intervention in severe stroke patients. Furthermore, this model facilitates a shift in stroke risk management from a “disease-centered” to a “patient-centered” approach, potentially improving patient outcomes, optimizing healthcare resource allocation, and reducing societal burden.

## Data Availability

The raw data supporting the conclusions of this article will be made available by the authors, without undue reservation.
